# Genome-wide methylation patterns from canine nanopore assemblies

**DOI:** 10.1093/g3journal/jkad203

**Published:** 2023-09-08

**Authors:** Peter Z Schall, Paige A Winkler, Simon M Petersen-Jones, Vilma Yuzbasiyan-Gurkan, Jeffrey M Kidd

**Affiliations:** Department of Human Genetics, University of Michigan, Ann Arbor, MI 48109, USA; Department of Computational Medicine and Bioinformatics, University of Michigan, Ann Arbor, MI 48109, USA; Department of Small Animal Clinical Sciences, College of Veterinary Medicine, Michigan State University, East Lansing, MI 48824, USA; Department of Small Animal Clinical Sciences, College of Veterinary Medicine, Michigan State University, East Lansing, MI 48824, USA; Department of Small Animal Clinical Sciences, College of Veterinary Medicine, Michigan State University, East Lansing, MI 48824, USA; Department of Microbiology and Molecular Genetics, College of Veterinary Medicine, Michigan State University, East Lansing, MI 48824, USA; Department of Human Genetics, University of Michigan, Ann Arbor, MI 48109, USA; Department of Computational Medicine and Bioinformatics, University of Michigan, Ann Arbor, MI 48109, USA

**Keywords:** de novo assembly, longread sequencing, Oxford Nanopore, methylation

## Abstract

Recent advances in long-read sequencing have enabled the creation of reference-quality genome assemblies for multiple individuals within a species. In particular, 8 long-read genome assemblies have recently been published for the canine model (dogs and wolves). These assemblies were created using a range of sequencing and computational approaches, with only limited comparisons described among subsets of the assemblies. Here we present 3 high-quality de novo reference assemblies based upon Oxford Nanopore long-read sequencing: 2 Bernese Mountain Dogs (BD & OD) and a Cairn terrier (CA611). These breeds are of particular interest due to the enrichment of unresolved genetic disorders. Leveraging advancement in software technologies, we utilized published data of Labrador Retriever (Yella) to generate a new assembly, resulting in a ∼280-fold increase in continuity (N50 size of 91 kbp vs 25.75 Mbp). In conjunction with these 4 new assemblies, we uniformly assessed 8 existing assemblies for generalized quality metrics, sequence divergence, and a detailed BUSCO assessment. We identified a set of ∼400 conserved genes during the BUSCO analysis missing in all assemblies. Genome-wide methylation profiles were generated from the nanopore sequencing, resulting in broad concordance with existing whole-genome and reduced-representation bisulfite sequencing, while highlighting superior overage of mobile elements. These analyses demonstrate the ability of Nanopore sequencing to resolve the sequence and epigenetic profile of canine genomes.

## Introduction

High-quality reference genome assemblies are an essential aspect for studies of genetic variation as well as efforts to define the genetic basis of phenotypes. Currently, a typical study identifies single nucleotide variation (SNV), and structural variation (SV) by comparing short-read sequencing data from a sample of interest to a high-quality reference (Formenti *et al.* 2022). Most commonly, the analyzed sample is sequenced using relatively short sequencing reads (e.g. Illumina paired reads on the order of 100–200 bp in length). Although highly accurate, correctly resolving repetitive, duplicated, and GC-rich sequences using this technology remains a challenge (Amarasinghe *et al.* 2020). With the advent of long-read sequencing technologies such as Pacific Biosciences (PacBio) and Oxford Nanopore Technologies (ONT), sequenced reads can now be routinely generated in the 10 s of kb range. This advancement has enabled the generation of genome assemblies with fewer gaps and increased representation of repeated regions (Amarasinghe *et al.* 2020).

Domestic dogs are a powerful system for genetic studies as recently reviewed (Ostrander *et al.* 2019). In fact, a current search of Online Mendelian Inheritance in Animals (OMIA) (Nicholas 2003) a catalogue of inherited disorders in animals, for entries for domestic dog traits and disorders, lists 888 phenotypes, 341 of which have a causal gene identified (https://www.omia.org; accessed 2023 July 16), leaving many others to be discovered. Dogs are the first domesticated species and have a unique population structure impacted by selection for specific traits and the establishment of defined breeds (Karlsson and Lindblad-Toh 2008), as well as other population bottlenecks due to factors such as wars and famine. This process has resulted in an over-representation of diseases and disorders in particular breeds such as copper-associated hepatopathy in Bedlington terriers, where studies have led to the identification of a novel gene in copper handling (Yuzbasiyan-Gurkan *et al.* 1997; van De Sluis *et al.* 2002), and retinitis pigmentosa in Papillons with *CNGB1* defects leading to the development of gene therapy translatable to humans (Winkler, Ekenstedt, *et al.* 2013; Petersen-Jones *et al.* 2018). Additionally, some diseases and disorders are prevalent in certain dog breeds that are comparatively rare in humans such as osteosarcoma in a number of large breeds of dog (Simpson *et al.* 2017), and HS in Bernese mountain dogs and flat-coated retrievers, allowing for genetic studies as well as translational drug discovery studies for dogs and humans. Long-read sequencing techniques offer a more complete view of genetic variation and may unlock breed-specific genetic features. To date, long-read genome assemblies have been published from 5 dog breeds, a Dingo, and a Greenland Wolf.

In this study, we have focused on the Bernese Mountain dog (BMD) and Cairn terrier (CT) genomes, both of which have interesting phenotypes with unresolved key genetic contributors. Bernese mountain dogs present with a great frequency of cancers (with 50% developing cancer) with over half developing histiocytic sarcoma (HS), a neoplasm that is rare both in many other dog breeds as well as in humans. In addition, the BMD fancy has been very progressive, maintaining an open database (the Berner-Garde Database, https://bernergarde.org/) as well as partnering with Michigan State University to establish a DNA and tumor repository. Cairn terriers have a high incidence rate of inherited ocular melanosis (OM) which is similar to human pigment dispersion syndrome. In Cairn terriers with OM, melanocytes proliferate and expand the iris root spreading to other parts of the uvea and sclera. Pigment and pigmented cells are shed into the aqueous humor, and impede aqueous drainage resulting in secondary glaucoma and vision loss (Petersen-Jones *et al.* 2007, 2008; Dawson-Baglien *et al.* 2019). Studies to date have failed to identify the genetic basis of these phenotypes, despite extensive genome-wide association studies (GWAS) for both conditions in both breeds: Shearin *et al.* (2012); Hedan *et al.* (2021) and exclusion of various genes (FANCG for BMD (Meek *et al.* 2022)) and various ones for CT OM (Winkler, Bartoe, *et al.* 2013). It is possible that specific aspects of the genome structure contribute to these disorders and make them challenging to identify with the approaches used to date.

The regulation of gene expression is critical for proper gene function. Whole-genome methylation analysis is critical for understanding the epigenetic regulation of the genome, modifications in gene expression, and cellular differentiation (Tirado-Magallanes *et al.* 2017). Current accurate methods to measure global methylation, such as reduced-representation bisulfite sequencing (rRBS) and whole-genome bisulfite sequencing (WGBS), have drawbacks that include cost and nonuniform sampling of genomic regions. Typically, methylated cytosines are identified by caustic bisulfite conversion followed by PCR amplification and Illumina sequencing. As previously discussed, short-read technologies have difficulty aligning to repeat-rich regions such as mobile elements. As their name implies, mobile elements are DNA sequences that can move within the genome, thereby impacting gene function and expression. Mobile elements are found in all organisms, and constitute a significant proportion of the genome in many species, including humans and dogs (Wells and Feschotte 2020). DNA methylation is a defense used to silence expression of mobile elements to restrict their proliferation (Zhou *et al.* 2020). Throughout evolution, mobile element sequences have been coopted to regulate host genes, and DNA methylation of mobile elements can alter the expression of the associated gene (Chuong *et al.* 2017). Global changes in mobile element methylation are also a hallmark of the epigenetic dysregulation found in some cancers (Grundy *et al.* 2022). Methylation can be inferred from alterations in the raw signal used in ONT sequencing, allowing for the characterization of methylation across mobile elements and other repeat regions.

In this study, we describe de novo assemblies of 4 breed dogs, including 3 that we sequenced using ONT technology and a reanalysis of existing read data from an additional individual. A systematic comparison with existing published canine assemblies confirms the high-quality of these new assemblies. Finally, we perform a genome-wide analysis of methylation using the raw ONT data we generated and compare genome-wide patterns with 2 existing publicly available WGBS and 57 rRBS datasets.

## Material and methods

### Sample selection, preparation, and sequencing

Two Bernese Mountain Dogs, BD (8-year-old female) and OD (10-year-old male), and a CT CA611 (12-year-old male) were selected for inclusion in this study. Fresh whole blood was collected from CA611 in a clinical setting and DNA extraction was performed the same day, while BD and OD blood samples were stored at −80°C. DNA was extracted from blood samples using a commercial DNA extraction kit with a modified protocol (Qiagen Sciences, Germantown, MD). Briefly, a red blood cell lysis buffer (0.32 M sucrose, 10 mM Tris, 5 mM MgCl2) was added in a 2-step fashion to whole blood (3× volume and then 2× volume, respectively). Cell lysis solution (Qiagen Sciences, Germantown, MD) was added to lyse the white blood cells followed by the addition of protein precipitation solution (Qiagen Sciences, Germantown, MD), isopropanol DNA precipitation, and a 70% ethanol wash step.

Short-read sequencing was conducted at ∼30× coverage (Novogene Co. Ltd) using the Illumina Novaseq6000 platform to generate paired-end 150 bp reads prepared with the NEBNext Ultra II DNA Library Prep Kit for Illumina Kit. High molecular weight DNA was shipped to the University of California Davis campus for long-read sequencing on the PromethION 19.01.6 platform using the SQK-LSK109 1D ligation chemistry kit.

### Genome assembly

**Fig. 1.**
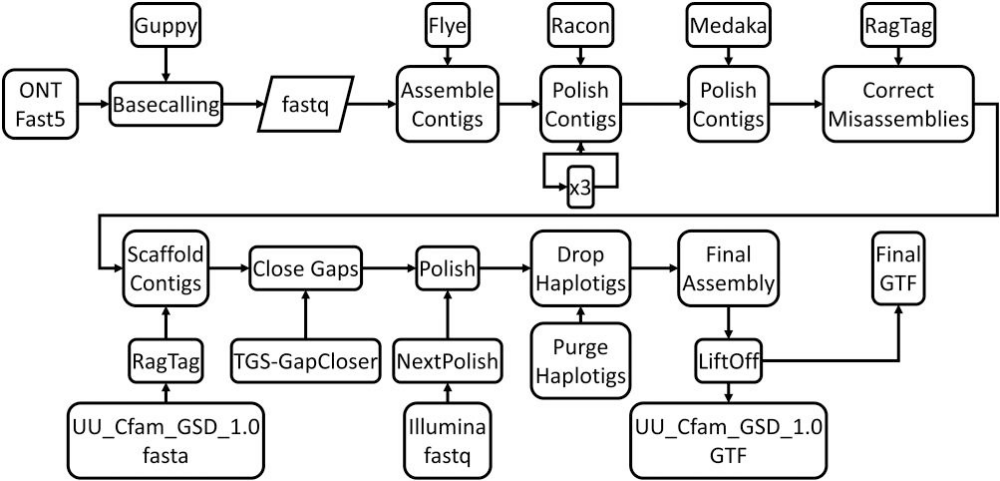
De novo genome assembly pipeline. The flow of analysis de novo genome assembly with an input of raw Oxford Nanapore Fast5 file format. The pipeline consists of 10 individual steps: (1) Basecalling converting Fast5 to fastq format with Guppy, (2) initial assembly of contigs with Flye, (3) 3 rounds of polishing with Racon, (4) additional contig polishing with Medaka, (5) correction of misassemblies with RagTag, (6) scaffolding of contigs to UU_Cfam_GSD_1.0 (Mischka) with RagTag, (7) closing of assembly gaps with TGS-GapCloser, (8) polishing using Illumina data with NextPolish, (9) removal of haplotigs with Purge Haplotigs, and (10) generation of GTF file with LiftOff.

A schematic detailing the pipeline utilized for the generation of the canine genome assemblies can be found in Fig. 1. Basecalling of the BD, OD, and CA611 samples, converting the raw ONT signal data from Fast5 to fastq format, was conducted using GuppyGPU (v5.0.11) (Wick *et al.* 2019) with the configuration file *dna_r9.4.1_450bps_hac_prom.cfg*. In addition to 3 samples generated for this study (BD, OD, and CA611), the ONT fastq files for the Golden Retriever genome (Yella; SRS6657854) (Player *et al.* 2021) were retrieved for reprocessing. The reprocessed Yella genome will hereafter be referenced as Yella_v2 and the original assembly as Yella_v1. The de novo assembly started with processing the fastq files with Flye v2.9 (RRID:SCR_017016) (Kolmogorov *et al.* 2019) for the initial construction of the assembly, using standard settings. This was followed by 3 rounds of assembly polishing with standard settings with Racon v1.5.0 (RRID:SCR_01642) (Vaser *et al.* 2017). Sequence correction was applied with Medaka v1.6.1 (https://github.com/nanoporetech/medaka), followed by assembly correction with RagTag v2.1.0 (Alonge *et al.* 2019). Scaffolding of the assembly to the CanFam4 German Shepherd genome (UU_Cfam_GSD_1.0, GCF_011100685.1) (Wang *et al.* 2021), supplemented with 3 Y chromosome sequences from a Labrador Retriever (ROS_Cfam_1.0, GCF_014441545.1), was conducted using RagTag. Closure of assembly gaps was completed using TGS-GapCloser v1.0.1 (RRID:SCR_017633) (Xu *et al.* 2020), with standard settings. Final polishing of the assembly with short-read Illumina data was conducted using NextPolish v1.4.1 (Hu *et al.* 2020) using standard settings.

Genome regions with a high-degree of heterozygosity can pose problems during assembly construction resulting in the generation of 2 individual primary contigs, a primary and its associated haplotig. The presence of allelic sequences in separate contigs can confound downstream analyses. To resolve this issue, the assemblies were further processed to remove contigs with unusual sequence coverage using the package Purge_Haplotigs v1.1.2 (RRID:SCR_017616) (Roach *et al.* 2018). Cutoffs of low, mid, and high points of read coverages were determined from visual inspection that was produced from produced from *readhist* function of Purge_Haplotigs. An average of 213 haplotigs were removed from each assembly. Gene annotations in gene transfer format (GTF) were converted from UU_Cfam_GSD_1.0 coordinates to each assembly using Liftoff v1.6.3 (Shumate and Salzberg 2020).

### Read and assembly quality control statistics

Assessment of long-read quality metrics was conducted using Nanostat v1.4.0 (De Coster *et al.* 2018), and short-read metrics were tabulated using Fastqc v0.11.5 (http://www.bioinformatics.babraham.ac.uk/projects/fastqc). Summary statistics of de novo genome assemblies were generated using QUAST v5.2.0 (RRID:SCR_001228) (Gurevich *et al.* 2013). Quantitative measurement of assembly annotation was conducted with Benchmarking Universal Single-Copy Orthologs (BUSCO) v5.4.3 (RRID:SCR_015008) (Simao *et al.* 2015) against the *carnivora_odb10* dataset. Additionally, 9 additional publicly availably long-read based canine genome assemblies (German Shepherd Dog: Nala (Field *et al.* 2020) and Mischka (Wang *et al.* 2021), Great Dane: Zoey (Halo *et al.* 2021), Boxer: Tasha (Jagannathan *et al.* 2021), Golden Retriever: Yella_v1 (Player *et al.* 2021), Basenji: Wags and China (Edwards *et al.* 2021), Greenland Wolf: mCanLor (Sinding *et al.* 2021), and Dingo: Sandy (Field *et al.* 2022)) were subjected to both QUAST and BUSCO interrogation to enable cross-genome comparisons. An additional metric of genome completeness was constructed using the software package Merqury v1.3 (RRID:SCR_022964) (Rhie *et al.* 2020). This method utilizes corresponding short-read Illumina data to compare k-mers found within the Illumina data to k-mers present in the assembled genome. Two metrics were utilized: k-mer completeness and Consensus Quality (QV). K-mer completeness is defined as the number of k-mers in the Illumina reads that are also found within the assembly. The QV estimates the frequency of consensus errors found in the assembly. Tabulation of included genome assemblies and associated quality control (QC) results can be found in Supplementary Table 1. QUAST statistics (number of contigs and N_50_ scores) at each step of the de novo assembly pipeline for BD, OD, CA611, and Yella_v2 are listed in Supplementary Table 2.

### Identification of SNV differences between genome assemblies

To further investigate global differences between genome assemblies, we identified single nucleotide variants (SNVs) among assemblies. The autosomes from each assembly were aligned to UU_Cfam_GSD_1.0 using minimap2 (RRID: SCR_018550, v2.20) (Li 2018), with the additional flags of *-c –cs*, outputting differences in *Pairwise mApping Format (PAF)* format. Resultant *PAF* data was sorted by chromosome and position, and variants were called using paftools from minimap2, with the flags *-l1000 -L1000*, outputting data in *Variant Call Format (VCF)*. VCF files were merged using BCFtools (RRID:SCR_005227, v1.12) (Danecek *et al.* 2021), with the additional flags, *–missing-to-ref -m snps,* limiting to only biallelic SNPs. The merged VCF file was piped to PLINK (RRID:SCR_001757, v1.9.0) (Purcell *et al.* 2007), generating PLINK browser extensible data (BED) format and calculating a principal component analysis (PCA). Application of PLINK SNV for PCA calculation included exclusion filters for genotyping rate (90%) and minor allele frequency (5%), resulting in 7,285,215 SNVs. A second PLINK BED format file was generated without applying filtering on genotyping rate and minor allele frequency for the purpose of distance calculations (SNVs = 14,002,038). PLINK BED format files were read into the R package SNPRelate (RRID:SCR_022719, v1.30.1) (Zheng *et al.* 2012). A distance calculation was conducted using the function *snpgdsDISS*. The distance matrix was utilized for the construction of a dissimilarity heatmap, and of a neighbor-joining tree via the *nj* function from the R package ape (RRID:SCR_017343, v5.7) (Paradis and Schliep 2019). Resultant tree data was ported to the R package ggtree (RRID:SCR_018560, v3.4.4) (Yu 2020) and rooted the tree with mCanLor as the outgroup. The PCA data from PLINK was separately imported into R for the generation of a PCA plot.

### Methylation analysis

Methylation calling from ONT reads was performed using Megalodon v2.5.0 (https://nanoporetech.github.io/megalodon) with UU_Cfam_GSD_1.0 serving as the reference genome. Fast5 files are utilized as the input to Megalodon, wherein basecalling is completed with Guppy, and modified bases are called using Remora v1.1.1 (https://github.com/nanoporetech/remora). Guppy basecalling utilized the same configuration files as previously (*dna_r9.4.1_450bps_hac_prom.cfg*). The *remora-modified-bases* flags were as follows: *dna_r9.4.1_e8 fast 0.0.0 5hmc_5mc CG 0*. The resultant outputs of the Megalodon software included read mapping in binary alignment map (BAM) format, modified base calling in bedmethyl format.

To ascertain the methylation status of different genetic features, the coordinates of exons, introns, promoters, CpG islands and shores, and 3′ and 5′ UTRs were extracted from the UU_Cfam_GSD_1.0 GTF file and saved in BED format. The RepeatMasker track was retrieved from the UCSC Genome Browser (RRID:SCR_005780) (Meyer *et al.* 2013) and the coordinates of long interspersed elements (LINEs), short interspersed elements (SINEs), and endogenous retroviruses (ERVs), were extracted. Putative LINEs were limited to those of at least 4 kb in length, SINEs between 150 and 250 bp, and ERVs at least 400 bp. The methylation state of CpG motif basepairs within the above-defined regions was extracted with the function *segmeth* from the software package methylartist (v1.2.6) (Cheetham *et al.* 2022).

For comparison of the ONT-based sequencing, the European Nucleotide Archive (RRID:SCR_006515) (Leinonen *et al.* 2011) was queried for canine methylation sequencing. Two WGBS datasets were identified: German Shephard Nala (Field *et al.* 2020) and Dingo Sandy (Field *et al.* 2022). A rRBS dataset (Janowitz Koch *et al.* 2016) of 57 breed dogs was also identified. The fastq files for each dataset were retrieved. The WGBS data was aligned to UU_Cfam_GSD_1.0 with abismal (v3.1.1) (de Sena Brandine and Smith 2021) using standard settings, resultant Sequence Alignment/Map (SAM) alignments were converted and sorted to BAM with SAMTools. Methylation calls were extracted to Bismark (RRID:SCR_005604) (Krueger and Andrews 2011) format with the function *extract* from the software package MethylDackel (v0.6.1) (https://github.com/dpryan79/MethylDackel), using the following flags: *–minOppositeDepth 5 –maxVariantFrac 0.5 –OT 20,148,20,120 –OB 25,145,25,145*. The MethylDackel output was further converted to the CGmap format with CGmapTools (v0.1.2) (Guo *et al.* 2018). The rRBS fastq files were aligned to UU_Cfam_GSD_1.0 using Bowtie2 (RRID:SCR_016368) (Langmead and Salzberg 2012) within the BSseeker2 (v2.1.8) (Guo *et al.* 2013) software package, using standard settings. Methylation calls for the rRBS data were extracted from the *bs_seeker2-call_methylation.py* function. The methylation state of basepairs, for both WGBS and rRBS datasets, within the previously defined genomic regions was extracted using the *mtr* function from CGmapTools. Hypomethylation calls (average methylation <= 20%) of specific genomic features (e.g. LINEs, SINEs, exons, etc.) were further filtered to require at least 3× coverage, ensuring their validity. Study/sample information and summary statistics of included methylation datasets can be found in Supplementary Table 4.

## Results

### Genome assembly results

We generated de novo assemblies of BD, OD, and CA611 from 4.6 M, 1.2 M, and 3.3 M ONT reads, respectively, with an average N_50_ read length of 24,739.3 bp (Table 1 and Supplementary Table 1). Following processing using a standardized pipeline (Fig. 1), the 3 assemblies consisted of an average of 651 contigs and an N_50_ average of 24.6 Mb (Table 1). The completeness of each assembly was evaluated with BUSCO (Simao *et al.* 2015) using the *carnivora_odb10* database, revealing that all 3 assemblies garnered a BUSCO complete score of at least 95%. Analysis of Illumina reads derived from the same individuals resulted in a mean k-mer completeness of 95.38% and a mean QV value of 95.38 (Table 1).

**Table 1.** Canine genome assembly information and statistics.

Assembly	Platform	Breed	Sex	# Reads	# Contigs	N50	QV	Kmer Completeness	BUSCO Complete*^a^*	BUSCO Duplicated	GTF Genes
BD	ONT PromethION	BMD	F	4,613,709	558	27,420,859	36.80	97.10	95.2% (97.5%)	2.29%	36,469
OD	ONT PromethION	BMD	M	1,232,008	1,013	11,505,439	30.00	95.30	95.2% (97.4%)	2.43%	36,423
CA611	ONT PromethION	CT	M	3,292,127	350	33,579,845	31.10	95.20	95.4% (97.7%)	2.68%	36,519
Yella_v2	ONT GridION	Labrador Retriever	M	7,726,029	681	25,750,950	30.90	93.90	95.3% (97.5%)	2.41%	36,504
Yella_v1	ONT GridION	Labrador Retriever	M	7,726,029	66,038	91,753	51.73	95.65	93.6% (95.8%)	2.38%	36,196
Mischka	PacBio Sequel	German Shepherd	F	34,084,767	2,801	14,840,767	35.95	96.85	95.3% (97.6%)	2.82%	43,427
mCanLor	PacBio HiFi	Greenland Wolf	M	7,188,959	248	34,375,412	59.04	96.42	95.5% (97.7%)	2.55%	31,191
Wags	PacBio Sequel	Basenji	M	16,407,741	3,630	3,131,423	34.99	96.39	93.6% (95.8%)	2.75%	24,339
China	ONT PromethION	Basenji	F	9,318,054	617	37,759,230	38.57	97.58	95.3% (97.5%)	2.43%	41,266
Nala	PacBio Sequel, ONT PromethION	German Shepherd	F	17,420,415	715	20,914,347	39.02	95.20	95.4% (97.6%)	2.51%	29,231
Tasha	PacBio PacBio RS II	Boxer	F	8,468,235	1,646	27,487,084	35.76	94.56	94.2% (96.4%)	2.32%	28,116
Zoey	PacBio PacBio RS II	Great Dane	F	23,639,954	1,726	4,722,218	37.24	96.61	94.6% (96.9%)	2.40%	24,090
Sandy	PacBio Sequel, ONT PromethION	Dingo	F	21,958,812	229	40,716,615	38.50	93.98	95.4% (97.6%)	2.53%	24,208

BUSCO complete values are listed with standard output followed by manual BLAST of joint missing BUSCO entries per methods.

In addition to the 3 newly assembled genomes, we explored the impact of software advancements on ONT-based genome assembly by reprocessing the previously released Yella genome assembly (Player *et al.* 2021). This assembly was selected due to the comparatively low N_50_ score (0.092 Mb) and a large number of contigs (*n* = 66,038). Yella was sequenced using the ONT platform and the initial assembling of contigs was conducted using minimap2 and miniasm (Li 2018). The ONT and Illumina fastq files were retrieved and processed through the same pipeline utilized for BD, OD, and CA611. The resultant genome assembly shows a substantial increase in continuity with an N_50_ of 25.8 Mb, and a total of 681 contigs. K-mer completeness between assemblies was similar (Yella_v1 = 95.7% vs Yella_v2 = 93.9%) while QV decreased from 51.73 to 30.9, and the BUSCO score increased from 93.5 to 95.3%. The decrease in the QV value for the reprocessed Yella genome can be largely attributed to the increased scrutiny of removing haplotigs and low-coverage contigs.

For OD, BD, CA611, and Yella_v2, the software package Liftoff (Shumate and Salzberg 2020) was utilized to map the annotations of UU_Cfam_GSD_1.0, generating assembly GTF files. Each of the resultant GTF files contained on average 36,479 genes (Table 1). Additionally, GTF files were retrieved for the other public assemblies from either Ensembl or NCBI. Only Yella_v1 lacked an available GTF file, therefore, Liftoff was utilized as previously described. Across the 9 publicly available genome assemblies, the GTF files contained an average of 31,340 genes (range: 24,090–43,427) (Table 1).

### Comparison to existing canine long-read assemblies

Since 2019, a total of 9 long-read based (mixture of PacBio and ONT) canine genome assemblies have been published, covering domestic dog *Canis lupus familiaris* (*n* = 7), wolf *Canis lupus* (*n* = 1), and dingo *Canis lupus dingo* (*n* = 1). Excluding Yella_v1 due to it being reprocessed in this study, the remaining 8 genome assemblies were likewise interrogated for the same quality metrics as a comparison to the newly minted BD, OD, and CA611. The mean number of reads utilized to generate these assemblies was 17.3 M (range: 7.2–34.1 M), where in the minimum is 3.0 M more than the average from BD, OD, and CA611. However, even with a fewer number of reads, the resulting assemblies N_50_ values were similar: a mean N_50_ value of 23 Mb (range: 3.1–40.7 Mb, median: 16.9 Mb) was calculated for these 8 assemblies, as compared to the 24.7 Mb mean calculated for BD, OD, and CA611. The k-mer completeness scores were likewise highly comparable: an average of 95.95% for the 8 public assemblies vs 95.38% for the 3 generated in this study.

BUSCO analysis, which measures genome completeness based on the presence of evolutionarily conserved genes, is commonly performed to assess the quality of a new genome assembly. We performed BUSCO analysis using the *carnivora_odb10* database, which is based upon data from 12 carnivores (cheetah, giant panda, domestic dog, domestic cat, Weddell seal, polecat, monk seal, walrus, leopard, tiger, and polar bear). The *carnivora_odb10* was established by identifying single-copy genes present in at least 90% of the input species, totaling 14,502 entries. The entries from the domestic dog were generated from the CanFam3.1 (Lindblad-Toh *et al.* 2005) genome assembly, totaling 14,429 entries mapping to 14,220 gene symbols. BUSCO scores for all assemblies were comparable. Surprisingly, we identified 440 entries that were missing from all assemblies. To explore these missing genes, we mapped BUSCO identifiers to gene symbols using OrthoDB (RRID:SCR_011980) (Waterhouse *et al.* 2013) and used basic local alignment search tool (BLAST) (RRID:SCR_004870, v 2.12.0) (Johnson *et al.* 2008) to search the amino acid sequences of the 440 entries against each assembly. At a threshold of 90% sequence identity, a total of 320 genes were confirmed in all assemblies (mean = 330), 15 were confirmed in at least one assembly, and 105 were not found in any assembly (Supplementary Table 3). Appending these manually confirmed BUSCO entries raised the percent of complete BUSCO entries by ∼2% per assembly (Table 1).

### Sequence similarity of genome assemblies

**Fig. 2.**
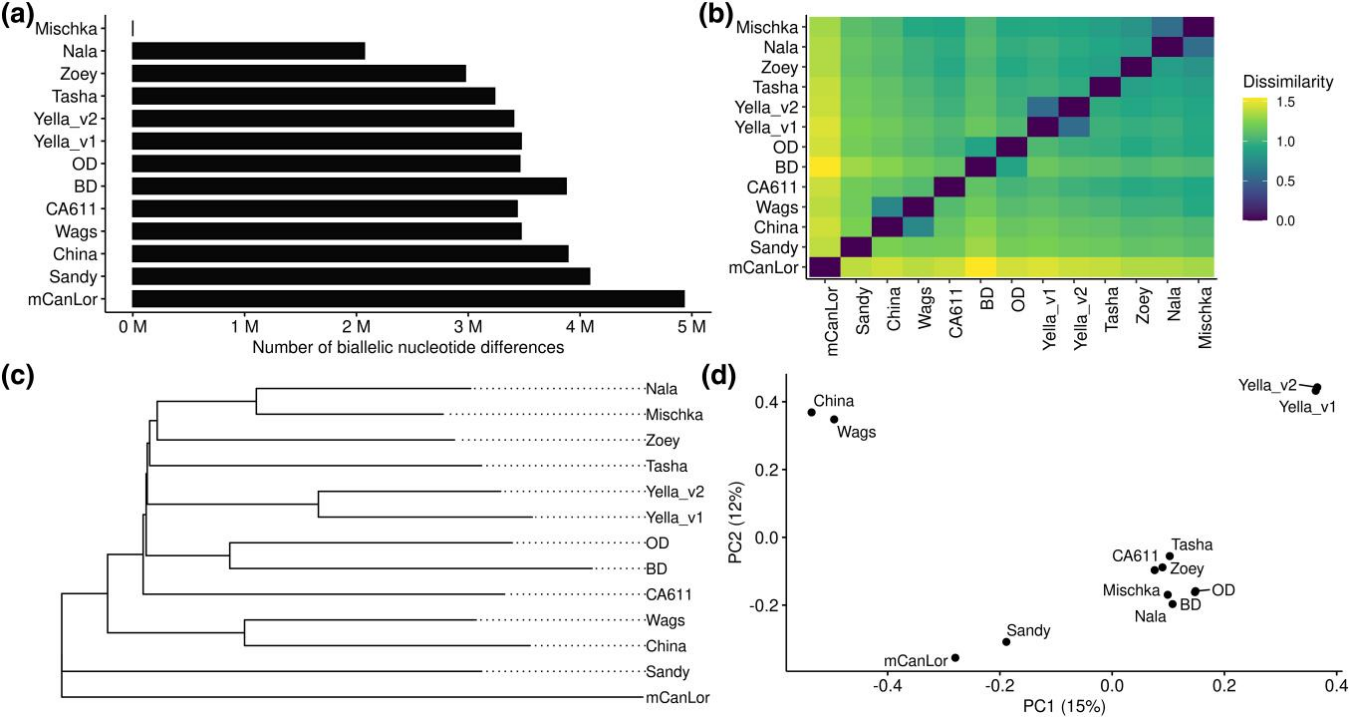
Biallelic SNP differences Among genome assemblies. Biallelic SNP differences were identified for all assemblies as compared to UU_Cfam_GSD_1.0 (Mischka). a) The total number of SNPs per assembly is given along x-axis, and assemblies are noted along y-axis. The self-comparison of Mischka-Mischka resulted in zero differences. b) A dissimilarity heatmap of all pairwise comparisons based on SNPs; darker shading denotes more similar samples, while lighter denotes more dissimilar. c) Consists of a neighbor-joining tree, rooted with mCanLor as the outgroup. d) Principal component analysis of SNPs across assemblies; x-axis denoted PC1 and y-axis PC2.

We identified biallelic SNV differences among the assemblies by aligning each to UU_Cfam_GSD_1.0. The number of identified SNVs reflects the expected relationship among samples. Excluding Mischka (the source of the UU_Cfam_GSD_1.0 assembly), the average number of SNPs was 3.53 M, with the fewest differences being found in the other German Shepherd Nala (*n* = 2.07 M). The greatest number of SNP differences were found in Greenland Wolf mCanLor (*n* = 4.93 M), followed by Sandy the Dingo (*n* = 4.01 M) (Fig. 2, Panel A). The pattern of allele sharing reinforced the wolf mCanLor as the most differentiated assembly while highlighting the clustering of like breeds (e.g. Wags and China: Basenji, OD and BD: Bernese Mountain Dogs) (Fig. 2, Panel B). Similarly, the construction of the neighbor-joining tree resulted in the placement of the German Shepherds (Mischka and Nala) as well as the Bernese Mountain Dogs and Basenjis (Fig. 2, Panel C) as sisters. The top 2 principal components (PCs) accounted for 15 and 12% of the variation, respectively, based on 7,285,215 SNVs. The principal component analysis resulted in the derivation of 4 groupings: (1) China & Wags (the basenji samples), (2) mCanLor & Sandy (wolf & dingo), (3) the 2 iterations of Yella, and (4) the remaining samples (Fig. 2, Panel D).

### Methylation results

**Fig. 3.**
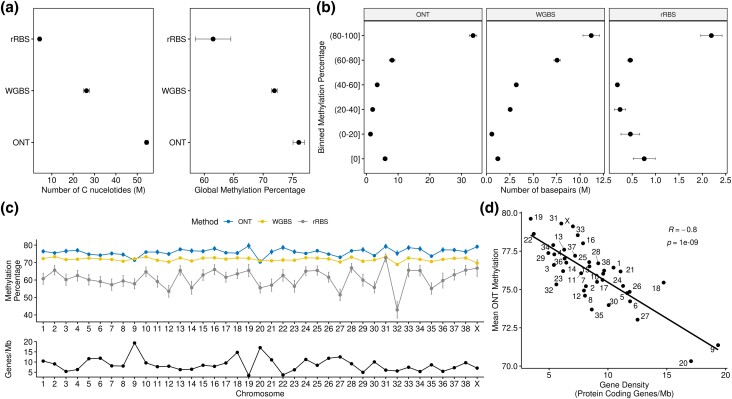
Genome-wide methylation summary. Methylation calls were conducted on whole blood samples from 3 nanopore (ONT) samples (BD, OD, and CA611), 2 whole-genome bisulfite (WGBS) samples (Nala, and Sandy), and 57 reduced-representation bisulfite (rRBS) breed dog samples. a) Two plots quantifying the total number of C nucleotides along x-axis and methylation protocol along y-axis (left) and, quantification of global methylation percentage along x-axis and protocol along y-axis (right). b) Quantification of methylation percentage into 6 bins along y-axis, sum of total basepairs per bin along x-axis, split by plots for each protocol. c) Quantification of methylation percentage by chromosome and protocol. The chromosome (autosomes + X) is along x-axis, and methylation percentage is along the y-axis. Color within plot denotes protocol: blue = ONT, yellow = WGBS, gray = rRBS. Gene density per chromosome is plotted below chromosomal methylation percentage, defined as number of protein-coding genes per megabase. d) Scatterplot consisting of chromosomal gene density along x-axis and mean ONT chromosomal methylation along the y-axis. Correlation between methylation and gene density was calculated using the Pearson method.

In parallel to the methylation analysis for BD, OD, and CA611, WGBS for Nala and Sandy, and rRBS dataset of 57 dogs were processed for comparison. The ONT datasets generated calls at an average of 54.4 Mb compared to 26.2 Mb for WGBS and 4.3 Mb for rRBS (Fig. 3, Panel A). The average global mean methylation percentage for the ONT samples was 76.1%, 71.9% for WGBS, and 61.5% for rRBS (Fig. 3, Panel A). When binning methylation levels, there is stark contrast in the average number of hypomethylated (<20%) basepairs: 1.1 Mb for ONT, 0.57 Mb for WGBS, and 0.46 Mb for rRBS (Fig. 3, Panel B). This was likewise found for hypermethylated basepairs (>=80%): 34.2 Mb for ONT, 11.2 Mb for WGBS, and 2.2 Mb for rRBS (Fig. 3, Panel B).

Splitting the methylation data by chromosome resulted in similar patterns of average methylation with the ONT samples having the highest average methylation, followed by WGBS and then rRBS (Fig. 3, Panel C). Sorting by average methylation per chromosome, chr31 was among the top 2 highest methylated across all 3 methodologies, while chr27 was in the lowest 2 methylated chromosomes (Supplementary Table 4). Comparing chromosomal methylation and gene density (defined as number of protein-coding genes per megabase) for the ONT datasets found a high-degree of negative correlation (R = −0.8, *P* = 1*10–9) (Fig. 3, Panel C).

**Fig. 4.**
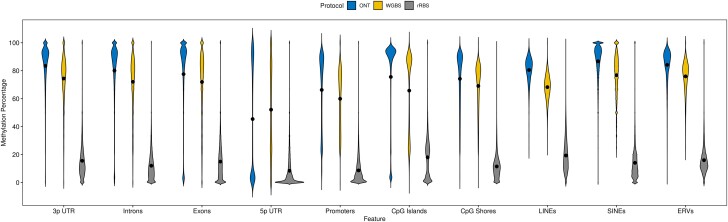
Percent methylation distribution of genomic features. Methylation calls were conducted on whole blood samples from 3 nanopore (ONT) samples (BD, OD, and CA611), 2 whole-genome bisulfite (WGBS) samples (Nala, and Sandy), and 57 reduced-representation bisulfite (rRBS) breed dog samples. Average methylation for individual genome features was extracted per sample. Specific genomic features are listed along the x-axis and the methylation percentage is along the y-axis. The interior of plot consists of violin plots denoting the spread of methylation by protocol: blue/left = ONT, yellow/middle = WGBS, gray/right = rRBS. Black dots denote the mean methylation per protocol and genomic feature.

The methylation data was further queried to ascertain the methylation status of different genomic features: exons, introns, promoters, 3′ and 5′ untranslated-regions (UTR), CpG islands and shores, ERVs, LINEs, and SINEs (Fig. 4). Exons, introns, 3′ UTR, CpG shores, ERVs, LINEs, and SINEs showed a bias of increased methylation while 5′ UTRs showed decreased methylation. Both promoters and CpG islands showed a bimodal distribution of methylation. Due to the decrease in the overall processed basepairs, the rRBS showed a decrease in the magnitude of methylated bases, regardless of the genomic feature. However, across all 3 protocols, 5′UTRs and Promotors displayed the lowest average level of methylation. The whole-genome protocols (ONT and WGBS) found the highest level of methylation in SINEs followed by 3′ UTRs. Comparing the distribution of genomic feature methylation across BD, OD, and CA611 showed a relative degree of similarity, however, CA611 displayed lower mean methylation across most features (Supplementary Fig. 1). This difference in average methylation could be attributed to a number of different factors including, but not limited to age, breed, or that BD and OD blood samples underwent −8°C storage as compared to the fresh collection of CA611 (Schroder and Steimer 2018).

**Fig. 5.**
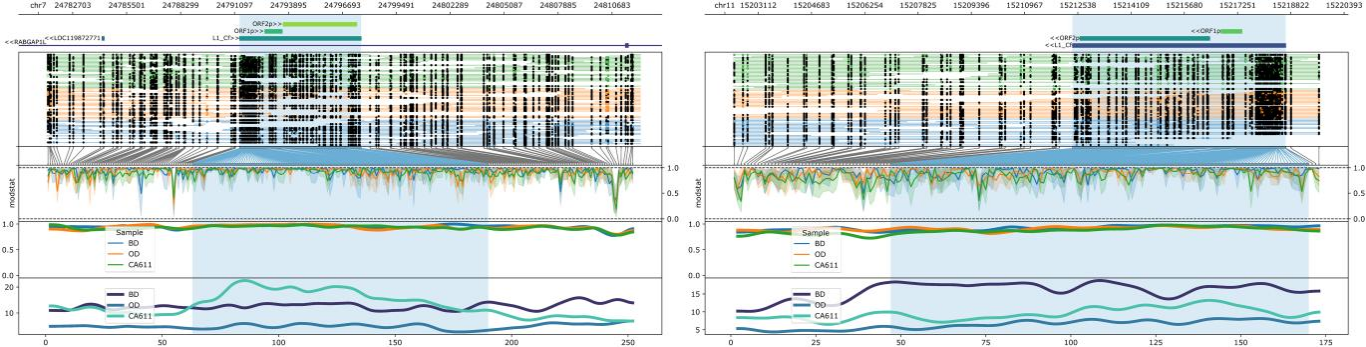
Hypemethylated LINEs located in intron or intergenic regions. Nanopore methylation results were queried for hypermethylated LINEs (≥90%) in BD, OD, and CA611. Resultant LINEs were further parsed for those with intact ORF1p and ORF2p and categorized into either intronic or intergenic regions. Methylation calls for the selected regions were plotted using Methylartist. The figure consists of 2 panels of hypermethylated LINEs, an intronic example on the left and intergenic on the right. Both panels have the same structure: genomic coordinates, genes, ORF1p, and ORF2p locations, read mappings with modified bases as circles (closed = modified) (open = unmodified), conversion from genome to CpG sites, raw log-likelihood ratios, smoothed methylation, and coverage. Within each panel, the region of the LINE sequence is highlighted in blue.

Mobile elements make important contributions to canine diversity (Wang and Kirkness 2005; Halo *et al.* 2021). Since the expression of mobile elements is correlated to the methylation state (Zhou *et al.* 2020), we investigated LINE methylation using the 3 ONT samples (BD, OD, and CA611). The ONT protocol was selected due to the increased coverage and methylation calls of the repeat-rich LINEs. A total of 5,671 LINEs (>=4 kb in length) that had at least 3× coverage in 3 samples were identified. We identified 181 hypermethylated LINEs (>=90% in all 3 samples) and no hypomethylated LINEs (<=20%). The resultant 181 LINEs were analyzed using BLASTX to identify intact putative open-reading frames for ORF1p (QOV08756.1) and ORF2p (QOV08757.1). LINE-1 retrotransposition, defined as the insertion of a LINE-1 sequence via an RNA intermediate, requires the function of both ORF1p and ORF2p. These 2 open-reading frames code for proteins necessary for functional retrotransposition (Moran *et al.* 1996). Of the 181 hypermethylated LINEs, 4.4% (*n* = 8) had intact ORF1p and ORF2p (>=99% identity) (Supplementary Table 5). Further interrogation of the 181 hypermethylated LINEs found 29 in intergenic regions, and 146 residing in introns of 131 total genes (Supplementary Table 5). The hypermethylated lines were filtered for those classified in the L1_Cf family, with intact open-reading frames, followed by selection of one residing within an intron and one in the intergenic region. The intronic LINE, located at chr7:24781363–24812150, resides within intron 14 of *RABGAP1L* (XM_038542442.1), while the intergenic LINE resides at chr11:15212380–15218668. Leveraging the ONT sequencing data allows for the complete characterization of the methylation signal along the length of these LINEs (Fig. 5).

## Discussion

In this manuscript, we present 2 BMD and one CT de novo genome assemblies based on ONT sequencing. Additionally, highlighting advancements in software, an updated reference assembly of the Yella Golden Retriever was produced with increased quality control metrics. These genome assemblies were compared to a plethora of published canine assemblies, followed up by a systematic assessment of quality control metrics calculated in a uniform manner (long-read QC, short-read QC, BUSCO, k-mer analysis, N_50_, number of contigs, etc.). At the time of writing, this is the first attempt to uniformly assess the quality of the 9 published long-read based canine assemblies.

According to the Earth Biogenome project's quantitative assembly standards for assembled genomes, N_50_ values (a measure of genome completeness) shall exceed 1 Mb, k-mer completeness >90%, and BUSCO complete values >90% (Lawniczak *et al.* 2022); these metrics have been exceeded for the included de novo assemblies. Indeed, the de novo assemblies of BD, OD, and CA611 resulted in highly competitive N_50_ values compared to the other published canine assemblies, while generated using the fewest number of reads. Another common metric for assessing genome assembly is the BUSCO score, which identifies the presence or absence of highly conserved genes. For the purpose of this study, we utilized the *carnivoa* database to uniformly investigate each assembly. While all assemblies had BUSCO complete percentages averaging 95% (range: 93.6–95.5%), we interestingly found that of the initial 14,502 BUSCO entries, 105 were not present in any assembly, even after manual interrogation. Removing these values from the calculation of BUSCO complete percentage, increased each assembly by an average of 2%. A similar issue highlighting the sensitivity of BUSCO analysis to alignment criteria has recently been described (Huang and Li 2023).

The included canine genomes were likewise compared utilizing genome-wide biallelic SNVs by comparing the assembled genomes to Mischka (UU_Cfam_GSD_1.0). The majority of the included genomes were generated from recognized dog breeds, with additional genomes from a Dingo and Greenland Wolf. These 2 genomes were correctly identified via the SNV analysis as the most divergent as it relates to the total number of SNVs and the pattern of allele sharing. Additionally, samples originating from like breeds (e.g. Mischka and Nala as German Shepherds, and China and Wags as Basenjis) resulted in their respective tight groupings via the dissimilarity, phylogenetic, and PCA analyses. These results were generated utilizing only the assembled genomes, a more thorough investigation using the associated longreads to fully categorize genome-wide SV is a desired next step.

An additional aspect of the ONT-based sequencing approach is the ability to call methylation status at a single basepair resolution. Ascertaining methylation status is an important step in understanding epigenetic regulation, specifically as it relates to repression of gene transcription, genomic imprinting, and the repression of mobile elements. Compared to common existing approaches such as reduced-representation and WGBS, the ONT method garners an increase in the number of identified methylated bases and overall methylation calls. These differences can, in-part, be attributed to the treatments required during the bisulfite treatment. To ascertain the methylation status, DNA is subjected to aggressive chemical treatment at a low pH and high temperature. This can lead to a high level of DNA degradation and thereby leading to an underestimation of methylation (Leontiou *et al.* 2015). This limitation is further magnified for rRBS-based approaches which, to limit sequencing to genomic regions of high CpG content, requires an additional step of restriction enzyme digestion (MspI as the most common enzyme of choice).

Regardless of the protocol underpinning the identification of methylation status, we found broad agreement in average genome-wide and chromosomal methylation percentage. Additionally, when focusing the analysis on specific genomic features, similar patterns arose across the methods. CpG islands displayed a bimodal distribution, 3′ UTRs having a higher average methylation compared to 5′ UTRs, and LINEs displaying a high level of methylation. Leveraging the ONT datasets allows for the ability to calculate methylation across even repeat-rich regions, a particular weakness of short-read bisulfite sequencing-based approaches.

The data herein furthers the field of canine genetics by (1) generation of de novo assemblies of 2 new breeds (BMD and CT) allowing for the further investigation of breed-specific diseases and genomic aspects, (2) highlighting the improvement of software technologies that warrant the revisiting and updating of existing genome assemblies (e.g. Yella), (3) demonstrating the applicability of using ONT based approaches for ascertaining genome-wide methylation.

## Supplementary Material

jkad203_Supplementary_Data

## Data Availability

Long-read nanopore sequencing and short-read Illumina sequencing have been deposited in the Sequence Read Archive under the BioProject PRJNA886700. The nanopore-based Whole-Genome Shotgun project has been deposited at DDBJ/ENA/GenBank under the accessions JARDRD000000000, JARDRE000000000, and JARDRF000000000. The versions described in this paper are versions JARDRD010000000, JARDRE010000000, and JARDRF010000000. The updated Yella_v2 Whole-Genome Shotgun project has been deposited at DDBJ/ENA/GenBank under the accessions DAOUOP000000000. The version described in this paper is version DAOUOP010000000. These current assembly versions and associated GTF files are available via figshare: https://doi.org/10.25387/g3.24019197. Supplemental material available at G3 online.
