# Correction to: Genome-wide methylation patterns from canine nanopore assemblies

**DOI:** 10.1093/g3journal/jkae099

**Published:** 2024-05-22

This is a correction to: Peter Z Schall, Paige A Winkler, Simon M Petersen-Jones, Vilma Yuzbasiyan-Gurkan, Jeffrey M Kidd, Genome-wide methylation patterns from canine nanopore assemblies, G3 Genes|Genomes|Genetics, Volume 13, Issue 11, November 2023, jkad203, https://doi.org/10.1093/g3journal/jkad203

An acknowledgement of funding support by the AKC Canine Health Foundation and Foundation of the Cairn Terrier Club of America has been added.

