# Supplementary material for: Genome-wide methylation patterns from canine nanopore assemblies
Source: G3 (Bethesda). 2023 Sep 8;13(11):jkad203. doi: 10.1093/g3journal/jkad203 (PMC10627269; doi:10.1093/g3journal/jkad203)
Supplement: jkad203_Supplementary_Data [file jkad203_supplementary_data.zip › Supplemental_Figure_Legend_G3-2023-404423.docx]

Supplemental Figure 1 - Percent Methylation Distribution of Genomic Features of ONT Samples

Methylation calls were conducted on whole blood samples from three nanopore (ONT) samples (BD, OD, and CA611). Average methylation for individual genome features were extracted per sample. Specific genomic features are listed along the x-axis and the methylation percentage along the y-axis. The interior of plot consists of violin plots denoting spread of methylation by sample: red= BD, orange=CA611, yellow=OD. Black dots denote the mean methylation per sample and genomic feature.
