# Supplementary figures and images for: Genome-wide methylation patterns from canine nanopore assemblies
Source: G3 (Bethesda). 2023 Sep 8;13(11):jkad203. doi: 10.1093/g3journal/jkad203 (PMC10627269; doi:10.1093/g3journal/jkad203)

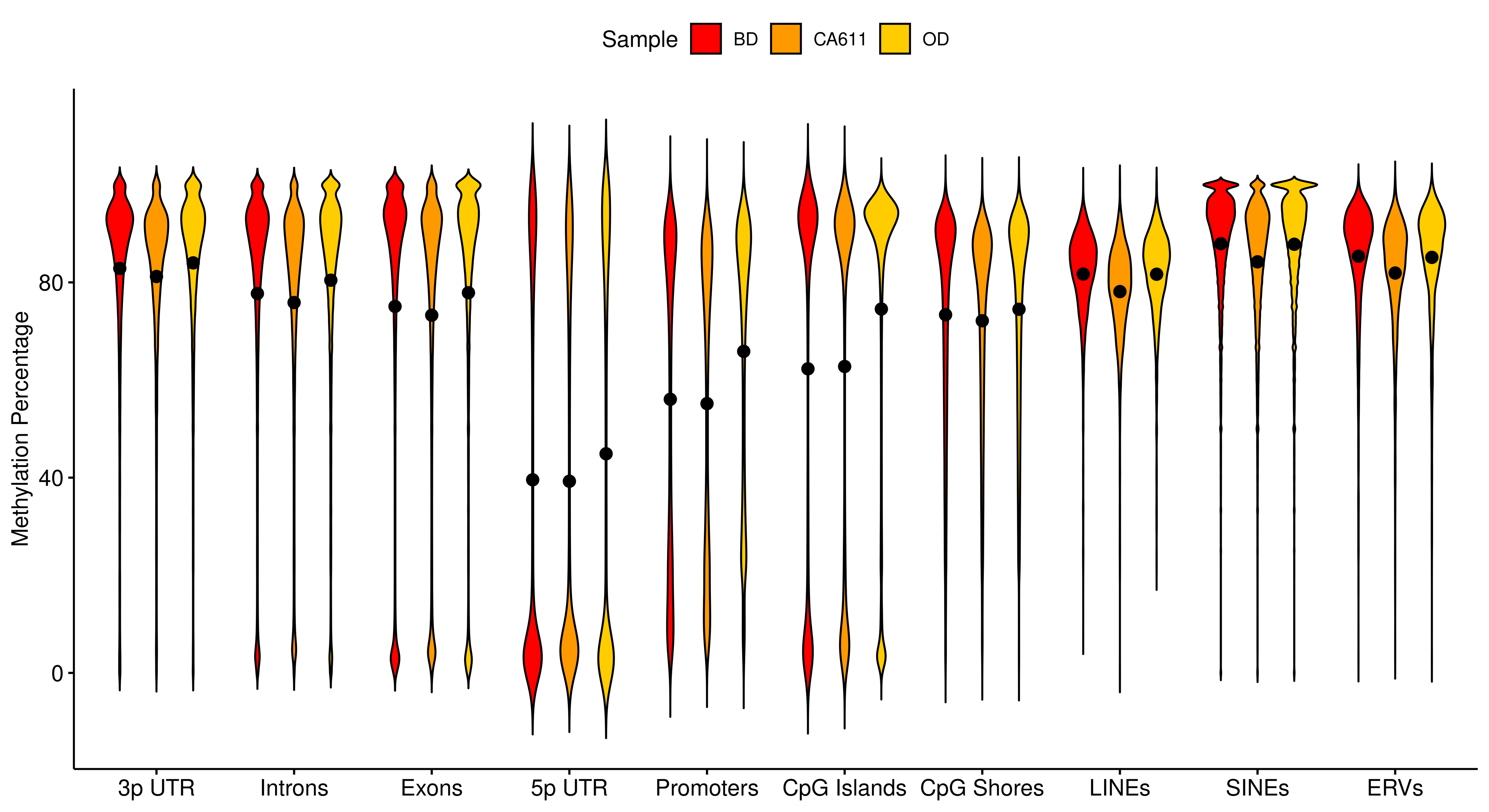

Supplement: jkad203_Supplementary_Data [file jkad203_supplementary_data.zip › Supplemental_Figure_S1_G3-2023-404423.tif]
